# The impact of altitude on early outcome following the Fontan operation

**DOI:** 10.1186/1749-8090-1-31

**Published:** 2006-10-02

**Authors:** Amir-Reza Hosseinpour, Catherine D Sudarshan, Paul Davies, Samer AM Nashef, David J Barron, William J Brawn

**Affiliations:** 1 Birmingham Children's Hospital, Steelhouse Lane, Birmingham, B4 6NH, UK; 2 Institute of Child Health, University of Birmingham, Whittall Street, Birmingham, B4 6NH, UK; 3 Papworth Hospital, Cambridge CB3 8RE, UK

## Abstract

**Background:**

The success of a Fontan circulation depends on several factors including low pulmonary vascular resistance. Pulmonary vascular resistance rises in response to hypoxia. Hypoxia is associated with altitude. Therefore, we wondered whether altitude is a risk factor for early failure after the Fontan operation. The aim was to test this hypothesis.

**Methods:**

Data were obtained from all published series of 'total cavopulmonary' Fontan operations since 1990. The early failure rate from each series and the altitude of the respective cities were recorded. Early failure was defined as death, takedown of Fontan, or transplantation during the same hospital admission. The association between altitude and failure rate was investigated by rank correlation and logistic regression.

**Results:**

24 series were identified from centres situated at altitudes ranging from sea level to 520 metres. The plot of failure rate versus altitude suggests that failure rate increases with altitude. Logistic regression did not fit the data adequately. This was possibly due to the influence of unmeasured and unknown factors affecting the results, as well as the fact that centres were not randomly chosen but were self-selected by virtue of publishing their results. However, Spearman's rank correlation was 0.74 (p = 0.001).

**Conclusion:**

The early outcome of the Fontan circulation appears to be adversely affected by altitude.

## Background

Low pulmonary vascular resistance (PVR) is a major criterion for the successful creation of a Fontan circulation [1]. It is a well known that PVR rises in response to hypoxia [2], which in turn correlates with altitude [3].

Therefore, we wondered whether altitude is a risk factor for early failure after completion of a Fontan circulation. The aim of this study was to test this hypothesis.

## Materials and methods

The data of all published series on the Fontan operation obtained from a literature search were reviewed. For each series, the number of patients treated, the primary diagnoses (case mix), the exact technique of the Fontan operation, the city where the procedures were performed and the early failure rate were recorded. Early failure was defined as death, takedown of the Fontan pathway, or heart transplantation during the same hospital admission. The altitude of the publishing centres, measured in metres above sea level and recorded to the nearest 5 metres, were obtained from the Royal Geographical Society and from the website [4].

Series that included patients operated on before 1990 were excluded for two reasons. The first was to reduce the impact of era on results. The second was to standardise the operations, since the procedure was still undergoing technical modifications in the 1980s. Therefore, only series incorporating the 'modern' Fontan completion techniques of total cavopulmonary connection by either lateral tunnel [5] or extra cardiac conduit [6] were included in the analysis.

The relationship between early failure rate of the Fontan circulation and altitude was analysed using the Spearman rank correlation test and binary logistic regression as implemented in the Egret statistical package (Cytel software, Massachussetts, USA).

## Results

24 series were identified to fulfil the inclusion and exclusion criteria [7-30]. They originated from centres with altitudes ranging from sea level to 520 metres. The centres, their altitudes in metres and their early Fontan failure rates are summarized in table 1.

**Table 1:** Cities where Fontan operations were performed.

**City**	**Altitude**	**n**	**Failure rate (%)**	**Reference**
Vancouver	5	30	3.3	6
Okayama	5	100	1	7
Fukuoka	5	100	0	8
Seattle	5	58	5	9
Boston	5	101	0	10
Tampa	5	45	0	11
Philadelphia	5	174	2.3	12
Los Angeles	5	100	0	13
Charleston	15	70	4.3	14
Kitakyushu	20	46	4.3	15
San Francisco	25	81	2.5	16
Berlin	50	73	6.7	17
Birmingham (UK)	90	103	2.9	18
Petah Tikvah (Israel)	105	40	7.5	19
Toronto	120	107	7.5	20
Dallas	135	49	0	21
Milwaukee	200	98	8	22
New Delhi	215	202	16.8	23
Kansas City	250	27	0	24
Atlanta	270	137	3.7	25
Ann Arbor	275	100	11	26
Nurnberg	315	37	8.1	27
Graz (Austria)	340	47	23.4	28
Santiago (Chile)	520	23	13	29

Cities where Fontan operations were performed, their altitudes(metres above sea level), early failure rate, total number of patient in the series (n), and the number of each series as it appears in the list of references.

One series exclusively dealt with hypoplastic left heart syndrome, and the right ventricle supported the systemic circulation [27]. In the remaining 23, the left ventricle supported the circulation in 50% to 70% of patients. The majority of the Fontan circulations in all the series were completed in stages: the patients had previously undergone a bi-directional superior cavo-pulmonary anastomosis or a hemi-Fontan procedure prior to completion. The practice of fenestration of the Fontan circuit varied greatly between the series.

**Figure 1:**
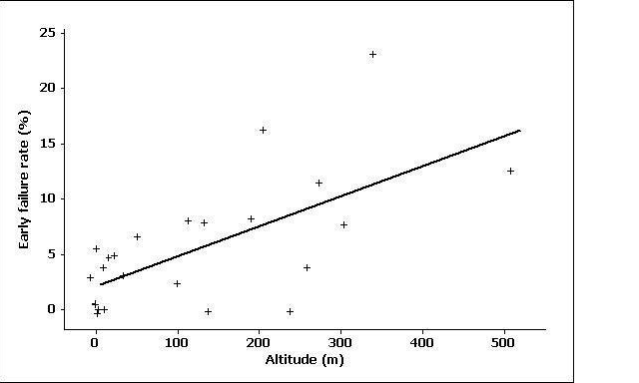
Graph representing the relationship between altitude and early Fontan failure rate in the respective centres. Each centre is represented by a +. The numbers as in the list of references have been omitted to avoid crowding.

Figure 1 shows the plot of failure rate versus altitude, and indicates that early failure rate increases with increasing altitude, with a Spearman rank correlation between failure rate and altitude of 0.74 (p = 0.001). Logistic regression analysis relating early Fontan failures to altitude was attempted, but this model did not fit the data adequately as shown by the Hosmer-Lemeshow lack of fit test (p = 0.001). This suggests that unmeasured or unknown factors also systematically influence the failure rate in a Fontan circulation.

## Discussion

The concern that altitude may have a negative impact on the outcome of the Fontan procedure is not new. Workers at Salt Lake City investigated the impact of altitude of the patients' home on long-term outcome [31]. They found that long-term mortality was not significantly affected, but noted a subjective reduction in exercise capacity at higher altitudes. The impact of altitude was also highlighted by the Houston group who reported a case of protein-losing enteropathy in a Fontan patient after relocating to a higher altitude [32]. This resolved upon moving back to a lower altitude.

Both atmospheric pressure and partial pressure of oxygen fall even with a modest rise in altitude [33] and this may increase PVR. The fall in atmospheric pressure is nearly linear within the range of altitudes that is relevant to most people (below 5000 metres), with a fall rate of 1% to 1.5% per 100 metres altitude above sea level. Therefore, even at the modest altitude of 500 metres, atmospheric pressure is 6.5% lower than it is at sea level. This reduction in atmospheric pressure results in a parallel reduction in the partial pressure of oxygen, since the proportion of oxygen in the atmosphere is constant (21%) up to an altitude of 110,000 metres [34]. Thus, at 500 metres the partial pressure of oxygen is also 6.5% lower than it is at sea level. Such a fall in oxygen levels may be sufficient to increase PVR. This is in keeping with our data, which suggests that the rate of early Fontan failure may increase at even modest altitudes, with a Spearman rank correlation of 0.74 (p = 0.001). The failure of logistic regression to account for all the non-random variation in failure rate is almost certainly due to factors beyond the scope of this study, such as individual surgeon and unit performance and variation in patient characteristics.

Some limitations of this study must be recognised. We have tried to minimize bias due to surgical technique by adhering to the contemporary series with comparable techniques of lateral tunnel or extra cardiac conduit Fontan circulations. These two techniques have similar outcomes according to the available evidence. However, several limitations persist. Although series do report the rate of Fontan takedown, it is not always clear whether this includes those that were taken down at the time of the original operation. Such a situation is rare, but it is possible that a surgeon may make such a decision when faced with problems and challenges at the time of surgery suggestive of early post-operative failure. It is possible that such cases may not be regarded as completed Fontan procedures and are therefore not included in the series. Clearly, this would result in under-reporting of the true failure rate. There is slight variation between the series as to whether the completion of the circulation was performed as a single or two-stage procedure but there is an even greater variation in the practice of fenestration of the Fontan conduit. These factors may bias the results. This study takes no account of patient selection, the skill and experience of the surgeon, the experience of the centre and the methods of post-operative management as these issues are rarely addressed in the published series. There are only 24 centres in this study and even fewer at higher altitudes. These may not be representative of all high altitude centres. Indeed, centres in this study were not randomly chosen but were self-selected by virtue of having published their Fontan results. Finally, pre-operative PVR of these patients prior to completion of the circulation is not available and therefore analysis of the relationship between PVR and altitude could not be performed.

Although the above limitations dictate a certain level of caution in the interpretation of the results, the data in this study suggest that the success of establishing a Fontan circulation may indeed be sensitive to altitude.
